# Participant retention in paediatric randomised controlled trials published in six major journals 2015–2019: systematic review and meta-analysis

**DOI:** 10.1186/s13063-023-07333-w

**Published:** 2023-06-14

**Authors:** Daisy M. Gaunt, Cat Papastavrou Brooks, Hugo Pedder, Esther Crawley, Jeremy Horwood, Chris Metcalfe

**Affiliations:** 1 Population Health Sciences, Bristol Medical School, University of Bristol, Canynge Hall, 39 Whatley Road, Bristol, BS8 2PS UK; 2 NIHR Applied Research Collaboration West, 9th Floor, Whitefriars, Lewins Mead, Bristol, BS1 2NT UK; 3 Bristol Trials Centre, Population Health Sciences, Bristol Medical School, University of Bristol, 1-5 Whiteladies Road, Bristol, BS8 1NU UK

**Keywords:** Systematic review, Meta-analysis, Paediatric, Randomised controlled trials, Attrition, Retention

## Abstract

**Background:**

The factors which influence participant retention in paediatric randomised controlled trials are under-researched. Retention may be more challenging due to child developmental stages, involving additional participants, and proxy-reporting of outcomes. This systematic review and meta-analysis explores the factors which may influence retention in paediatric trials.

**Methods:**

Using the MEDLINE database, paediatric randomised controlled trials published between 2015 and 2019 were identified from six general and specialist high-impact factor medical journals. The review outcome was participant retention for each reviewed trial’s primary outcome. Context (e.g. population, disease) and design (e.g. length of trial) factors were extracted. Retention was examined for each context and design factor in turn, with evidence for an association being determined by a univariate random-effects meta-regression analysis.

**Results:**

Ninety-four trials were included, and the median total retention was 0.92 (inter-quartile range 0.83 to 0.98). Higher estimates of retention were seen for trials with five or more follow-up assessments before the primary outcome, those less than 6 months between randomisation and primary outcome, and those that used an inactive data collection method. Trials involving children aged 11 and over had the higher estimated retention compared with those involving younger children. Those trials which did not involve other participants also had higher retention, than those where they were involved. There was also evidence that a trial which used an active or placebo control treatment had higher estimated retention, than treatment-as-usual. Retention increased if at least one engagement method was used. Unlike reviews of trials including all ages of participants, we did not find any association between retention and the number of treatment groups, size of trial, or type of treatment.

**Conclusions:**

Published paediatric RCTs rarely report the use of specific modifiable factors that improve retention. Including multiple, regular follow-ups with participants before the primary outcome may reduce attrition. Retention may be highest when the primary outcome is collected up to 6 months after a participant is recruited. Our findings suggest that qualitative research into improving retention when trials involve multiple participants such as young people, and their caregivers or teachers would be worthwhile. Those designing paediatric trials also need to consider the use of appropriate engagement methods.

**Research on Research (RoR) registry:**

https://ror-hub.org/study/2561

**Supplementary Information:**

The online version contains supplementary material available at 10.1186/s13063-023-07333-w.

## Introduction

Retention of participants in randomised controlled trials (RCTs) is an important issue currently concerning clinical trialists [1–3]. Several research projects are investigating retention in RCTs [4–6]; however, none specifically explore retention in paediatric trials. If there are specific factors which influence whether participants complete and return outcomes, and these either lead to a significant amount of missing data and/or a differential drop-out rate between treatment groups, then misleading conclusions may be drawn from the results [7, 8]. There are still fewer trials in paediatrics than in adults, potentially due to the increase in complexity of ethics for research involving children, fewer safety studies of clinical formulations and dosing regimens that vary with physical age [9], and therefore, it is important to minimise research wastage by designing trials that are likely to retain participants.

There are unique differences in the context and design of trials in paediatrics compared with those involving adults, such as outcome reporting by either/both the young person and their caregiver, and key developmental differences between young people across the age range in a trial, which mean that only some of the children are allowed to take part on their own without caregiver consent, and can therefore withdraw or be lost-to-follow-up without their caregivers’ knowledge [10].

Two recent systematic reviews and meta-analyses [11, 12] which investigated strategies to increase retention of participants, found few studies that involved children or young people. Gillies et al. [11] found only two retention trials explicitly involving young people; Greig et al. [13] which compared postal follow-up with clinic follow-up with low certainty of evidence and Marsh et al. [14] who used either telephone, postal or clinic follow-up with or without an incentive with very low certainty of evidence, and three that involved some young people as well as adults, Henderson et al. [15] used a lottery compared to usual follow-up with low certainty of evidence, Cook et al. (Cook JA, Bongard E, Heneghan C, Butler CC: SWAT 90 evaluation: Does the time at which a participant incentive is given affect the retention rate?, Unpublished) which used an conditional vs. unconditional monetary reward with very low certainty of evidence and Bailey et al. [16] who use postal follow-up requests plus two different monetary vouchers included vs. no follow-up requests or incentives, no comparison. El-Feky et al. [12] found only one study that included only young people; Ezell et al. used multi-faceted retention strategies [17], although it was not clear which of these strategies improved retention, and two studies that included young people and adults; Bailey et al. [16] used an online questionnaire and a shortened version of the online questionnaire by post; and Sellers et al. [18] used routine strategies (support groups, home visits) and intensive tracing efforts. A 2016 systematic review [19] of child and parent factors which influence recruitment and retention of participants to RCTs only included children from birth to 12 years. However, the authors were unable to meta-analyse the 28 included RCTs using any of the sociodemographic variables due to the variability in reporting. Kearney et al. [20] (published conference abstract) reviewed trials, not specifically paediatric, which were reported in *Journal of the American Medical Association* (JAMA), *New England Journal of Medicine* (NEJM), *British Medical Journal* (BMJ) and *The Lancet* in 2013 and 2018. In 2013, missing primary outcome data was associated with outpatient data collection, trials within chronic conditions, smaller trials (recruitment target and number randomised), shorter recruitment and longer follow up. A review of trials in published in six major journals between July and December 2004 by Torien et al. [21] found that the number of randomised treatment groups and treatment focus were associated with retention. Walters et. al [22] found that in a review of trials funded and published by the United Kingdom National Institute for Health Research Health Technology Assessment Programme, the setting of the trial, final target recruitment and total recruitment were associated with retention. Evidence for an association with retention was also found for these factors by Jacques et al. [23] in their review of trials published in the National Institute for Health Research Journals Library between 1997 and 2020, as well as the number of randomised treatment groups. None of these reviews specifically investigated or described whether the included trials were in paediatrics. In this review, we aim to investigate whether there are context or trial-specific factors which influence retention of participants in paediatric RCTs.

## Methods

We defined retention as “All randomised participants continuing in the trial and providing primary outcome data”. The primary outcome was chosen as we hypothesised that most trials would focus on the retention of participants until this was reported, as advised in the Consolidated Standards of Reporting Trials (CONSORT) reporting guidelines [24].

We sought reports of RCTs in paediatrics published in the following journals: NEJM, BMJ, JAMA, The Lancet, Paediatrics and JAMA Paediatrics. These were selected as we were interested in factors that impacted retention even in, we assumed, well-resourced, and well-designed trials, and so that the potential association between trial factor and retention may be more precisely estimated (low variation, narrow confidence intervals). Additional file 1 Appendix 1 reports the Medline database search strategy. The inclusion criteria were that the trial was a randomised controlled trial, which included a primary outcome measured for children aged under 18, and an intervention specifically targeting children (rather than, for example, a training intervention aimed at caregivers). If the trial recruited from both adult and paediatric populations, the retention data needed to be presented separately for paediatric participants. Factorial designs were not excluded. Excluded study designs and publication types included systematic reviews or meta-analyses, N-of-1 trials, follow-on trials or commentaries to the original RCTs, or conference abstracts.

Covidence systematic review software [25] was used to organise and store papers, and for data extraction. Two reviewers (DG and HP) reviewed titles and abstracts independently. All discrepancies were resolved through robust discussion. DG reviewed all full-text articles, and data extraction was carried out by DG and CPB. Initially, data were extracted from 10 papers by both reviewers and all discrepancies were discussed. After further clarification of the definition of the factors, data extraction continued independently. Additional file 1 Appendix 2 includes the data extraction proforma. No risk-of-bias assessments were used as these trials were published in peer-reviewed high-impact factor journals, attrition is a component of a risk-of-bias assessment, and other biases that are assessed in a risk-of-bias assessment would not affect the conclusion of this methodological study. The protocol was pre-specified (Additional file 1 Appendix 3), but not registered in PROSPERO as this methodological review compares the completion of the primary outcome by participants across trial factors, and does not address health outcomes. It was instead registered in the Research on Research (RoR) registry (https://ror-hub.org/study/2561).

The numbers of participants randomised, and retained, by randomised groups were extracted. If participants died during the trial and death was the primary outcome, they were counted as being retained. Retention for a trial was calculated as a proportion; participants reported as completing the primary outcome across all treatment groups divided by total participants randomised. All participants from all randomised groups within a multi-group trial were included.

As this review was designed to investigate differences between trials based on trial factors, a random effects meta-analysis of the proportion retained in each trial was used. All trials were to be included in every analysis.

A generalised linear mixed model (GLMM) with a binomial distribution and logit link was used [26]. To investigate potential sources of heterogeneity between trials, a univariate random-effects meta-regression analysis [27, 28] used each of the trial factors in-turn. Each was included as a categorical explanatory variable (*x*_*i*_), with *k* categories, in the link function.

The absolute proportion of participants retained was reported for each categorical explanatory variable with 95% confidence interval. As the aim of this review was not to predict retention in a future RCT the prediction intervals are not reported. The likelihood ratio test comparing the meta-regression model with, and without, the categorical explanatory variable was used to determine whether there was any evidence of this influencing retention. Due to the limited power to detect interactions between combinations of explanatory variables, these analyses were not investigated [29]. Analyses were pre-specified, except the post-hoc sensitivity analyses which were conducted to assess bias of the included trials.

Heterogeneity in retention across the reviewed studies was quantified using the τ^2^ statistic (between-trial heterogeneity), presented with a test of the null hypothesis of no heterogeneity.

All analysis was run in Stata (version 16.1) [30], using the metapreg command [31].

## Results

**Fig. 1:**
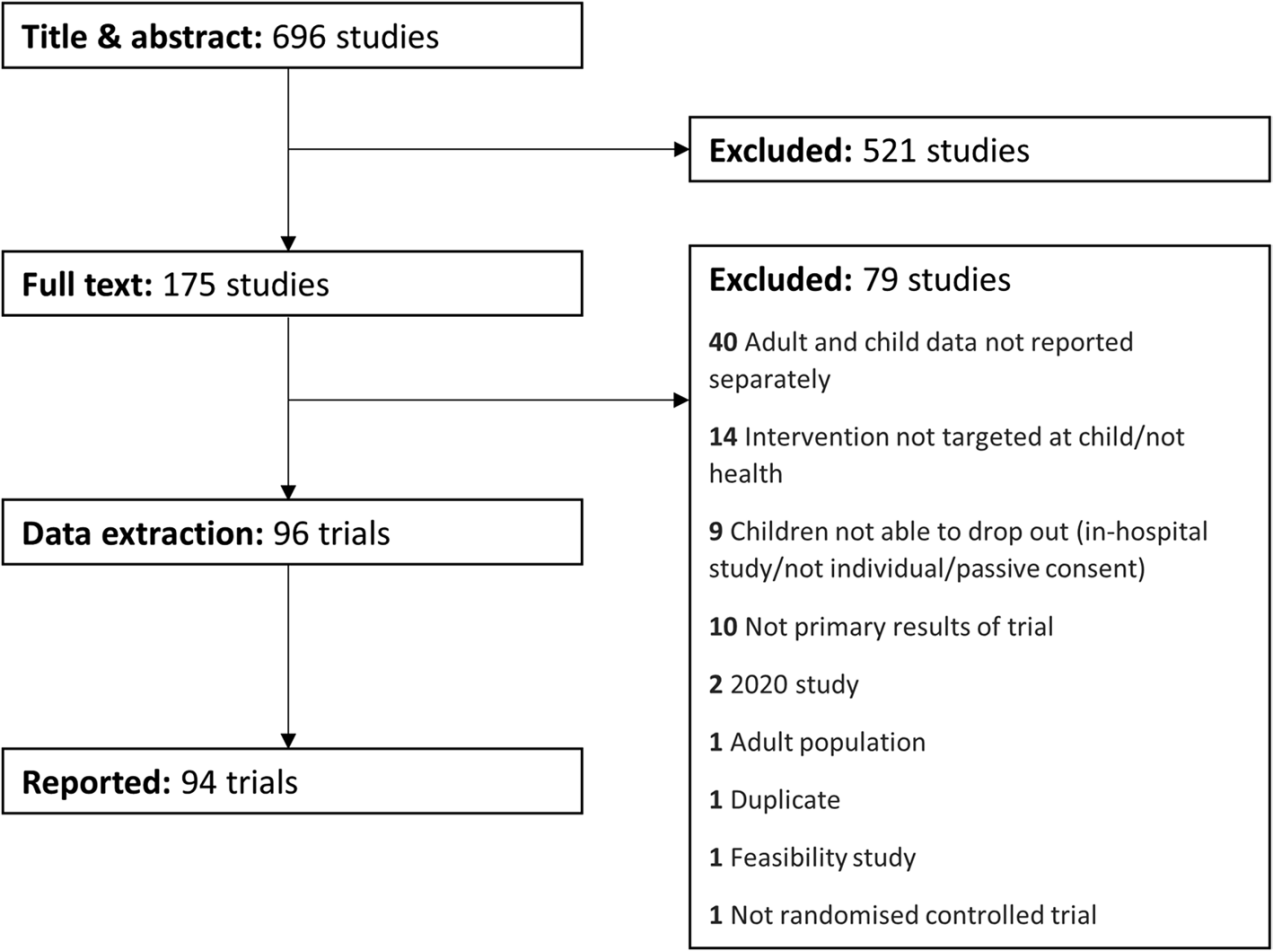
PRISMA flowchart

The literature search returned 684 papers on 24/01/2020, and 175 trials were included in the full-text review. Ninety-six trials were included after a full-text review in data extraction (PRISMA flowchart, Fig. 1). Ninety-four trials reported retention of the primary outcome, with two trials being excluded because no data were presented on the retention of the primary outcome. All data extracted from all 94 included trials are reported in Additional file 2, and all results reported are for 94 trials unless otherwise indicated.

Eighty-two trials had two treatment groups, nine had three and three had four treatment groups. Seventeen trials were published in 2015, 21 in 2016, 21 in 2017, 18 in 2018 and 17 in 2019. The journal that published the most was the *New England Journal of Medicine*, followed by *Paediatrics* (Table 1). Five trials used a factorial design.

**Table 1:** Results of meta-regression

Factor	Number of RCTs	Retention estimate (95% CI)	Likelihood ratio test *P*-value	τ^2^ (*p*-value)
**Journal**				
* NEJM*	33	0.97 (0.94, 0.98)	Not compared	
* Paediatrics*	23	0.93 (0.87, 0.97)		
* JAMA*	16	0.93 (0.85, 0.97)		
* Lancet*	13	0.95 (0.88, 0.98)		
* JAMA paediatrics*	8	0.87 (0.67, 0.96)		
* BMJ*	1	0.56 (0.54, 0.58)		
**Funding Source**			0.04	2.98 (< 0.001)
Government	46	0.92 (0.87, 0.95)		
Industry	24	0.96 (0.92, 0.98)		
Multiple funders	11	0.98 (0.94, 0.99)		
Third sector	8	0.98 (0.94, 0.99)		
Academic	8	0.87 (0.60, 0.97)		
**Population**			0.87	3.38 (< 0.001)
Clinical	68	0.95 (0.92, 0.97)		
General	26	0.94 (0.89, 0.97)		
**ICD-10 disease area (2019)**			0.46	2.83 (< 0.001)
IV Endocrine, nutritional, and metabolic diseases	13	0.92 (0.81, 0.97)		
X Diseases of the respiratory system	12	0.97 (0.92, 0.99)		
III Diseases of the blood and blood-forming organs and certain disorders involving the immune mechanism	10	0.97 (0.91, 0.99)		
I Certain infectious and parasitic diseases	10	0.96 (0.90, 0.99)		
VI Diseases of the nervous system	9	0.91 (0.76, 0.97)		
V Mental and behavioural disorders	8	0.85 (0.64, 0.95)		
XI Diseases of the digestive system	5	0.96 (0.86, 0.99)		
IX Diseases of the circulatory system	4	0.99 (0.93, 1.00)		
XII Diseases of the skin and subcutaneous tissue	4	0.90 (0.62, 0.98)		
XIII Diseases of the musculoskeletal system and connective tissue	3	0.97 (0.80, 1.00)		
XIX Injury, poisoning and certain other consequences of external causes	3	0.88 (0.51, 0.98)		
XVI Certain conditions originating in the perinatal period	3	0.88 (0.52, 0.98)		
XIV Diseases of the genitourinary system	2	0.97 (0.75, 1.00)		
XVIII Symptoms, signs and abnormal clinical and laboratory findings, not elsewhere classified	2	0.93 (0.57, 0.99)		
XV Pregnancy, childbirth and the puerperium	2	0.99 (0.90, 1.00)		
XXI Factors influencing health status and contact with health services	2	0.90 (0.47, 0.99)		
VII Diseases of the eye and adnexa or VIII Diseases of the ear and mastoid process	2	0.90 (0.47, 0.99)		
**Severity of condition**			0.33	3.31 (< 0.001)
Chronic	61	0.94 (0.91, 0.96)		
Acute	33	0.96 (0.92, 0.98)		
**Intervention aim**			0.51	3.33 (< 0.001)
Management of condition	44	0.95 (0.91, 0.97)		
Preventative	34	0.93 (0.88, 0.96)		
Curative	16	0.96 (0.91, 0.99)		
**Site**			0.78	3.38 (< 0.001)
Multi	81	0.95 (0.92, 0.96)		
Single	13	0.95 (0.88, 0.98)		
**Trial design**			0.34	3.35 (< 0.001)
Parallel group	91	0.95 (0.93, 0.96)		
Cross-over	3	0.87 (0.43, 0.98)		
**Total sample size**			0.34	3.33 (< 0.001)
< 1000	70	0.94 (0.91, 0.96)		
≥ 1000	24	0.96 (0.92, 0.98)		
**Number of treatment groups**			0.45	3.36 (< 0.001)
Two	82	0.95 (0.93, 0.97)		
Three or more	12	0.92 (0.81, 0.97)		
**Treatments**			0.41	3.15 (< 0.001)
Pharmacological	64	0.96 (0.93, 0.97)		
Behavioural change	13	0.93 (0.82, 0.97)		
Psychological therapy	5	0.91 (0.66, 0.98)		
Other medical procedure	4	0.89 (0.59, 0.98)		
Medical device	3	0.98 (0.85, 1.00)		
Other	3	0.78 (0.32, 0.96)		
Surgical procedure	2	0.98 (0.76, 1.00)		
**Control treatments (*n* = 91)**			0.05	3.12 (< 0.001)
Active	34	0.97 (0.95, 0.98)		
Placebo	33	0.94 (0.89, 0.96)		
Treatment-As-Usual	24	0.92 (0.84, 0.96)		
**Randomisation**			0.17	3.30 (< 0.001)
Individual	82	0.95 (0.93, 0.97)		
Cluster	12	0.90 (0.76, 0.96)		
**Age of youngest children**			0.04	3.10 (< 0.001)
0 +	58	0.96 (0.93, 0.97)		
4 +	18	0.94 (0.87, 0.97)		
7 +	12	0.84 (0.65, 0.93)		
11 +	6	0.98 (0.91, 1.00)		
**Additional participants**			0.04	3.25 (< 0.001)
None	31	0.97 (0.94, 0.98)		
Additional participants	63	0.93 (0.90, 0.96)		
**Intervention setting**			0.44	3.17 (< 0.001)
Home	43	0.94 (0.90, 0.96)		
Healthcare	33	0.96 (0.93, 0.98)		
School	7	0.94 (0.81, 0.98)		
Emergency department and home	3	0.98 (0.86, 1.00)		
Home and school/daycare	3	0.75 (0.28, 0.96)		
Research centre	3	0.91 (0.58, 0.99)		
Other	2	0.94 (0.56, 0.99)		
**Length of intervention**			0.07	2.87 (< 0.001)
In-hospital stay	11	0.98 (0.93, 0.99)		
Less than 1 month	13	0.97 (0.92, 0.99)		
Between 1 to 3 months (inclusive)	18	0.86 (0.74, 0.93)		
Over 3 to 6 months (inclusive)	16	0.96 (0.91, 0.98)		
Over 6 to 12 months (inclusive)	12	0.95 (0.87, 0.98)		
Over 12 months to 2 years (inclusive)	6	0.95 (0.82, 0.99)		
Over 2 years	4	0.79 (0.41, 0.95)		
Variable depending on treatment/trial design	12	0.97 (0.91, 0.99)		
Until cure	2	0.92 (0.51, 0.99)		
**Length of RCT**			0.19	3.14 (< 0.001)
Up to 6 months (inclusive)	28	0.97 (0.94, 0.98)		
Over 6 to 12 months (inclusive)	22	0.94 (0.88, 0.97)		
Over 12 months to 2 years (inclusive)	24	0.95 (0.90, 0.98)		
Over 2 years	15	0.88 (0.76, 0.95)		
Variable depending on treatment/trial design	5	0.93 (0.72, 0.98)		
**Total number of follow-up assessments**			0.40	3.08 (< 0.001)
One to four	37	0.94 (0.89, 0.96)		
Five or more	57	0.95 (0.93, 0.97)		
**Length of time to the primary outcome(s)**			0.01	2.95 (< 0.001)
Up -months (inclusive)	29	0.95 (0.91, 0.97)		
Over 6 to 12 months (inclusive)	22	0.93 (0.86, 0.97)		
Over 1 year	23	0.90 (0.81, 0.95)		
Variable depending on treatment/trial design	20	0.98 (0.96, 0.99)		
**Number of follow-up assessments before primary outcome(s)**			0.01	2.89 (< 0.001)
None	19	0.92 (0.84, 0.96)		
One to four	24	0.90 (0.81, 0.95)		
Five or more	35	0.95 (0.92, 0.97)		
Other	16	0.98 (0.96, 0.99)		
**Primary outcome data collection**			0.03	2.93 (< 0.001)
Trial-specific clinic visit	55	0.94 (0.90, 0.96)		
Call with/without survey	9	0.95 (0.86, 0.98)		
Hospital or routine data	9	0.99 (0.97, 1.00)		
Researcher visits participant	6	0.93 (0.78, 0.98)		
Survey	5	0.89 (0.63, 0.97)		
School visit	5	0.84 (0.90, 0.96)		
Other/multiple methods	5	0.97 (0.86, 0.99)		
**Primary outcome**			0.10	3.10 (< 0.001)
Single	41	0.93 (0.88, 0.96)		
Repeated measures over time	27	0.96 (0.92, 0.98)		
Composite	11	0.91 (0.78, 0.97)		
Time-to-event	15	0.98 (0.94, 0.99)		
**Primary outcome report**			0.11	3.14 (< 0.001)
Objective measurement	44	0.95 (0.92, 0.97)		
Assessor report	27	0.94 (0.89, 0.97)		
Teacher/caregiver report	11	0.91 (0.77, 0.97)		
Participant self-report	7	0.93 (0.77, 0.98)		
Multiple methods or routine data	5	0.99 (0.96, 1.00)		
**Number of other follow-up methods**			0.64	3.34 (< 0.001)
One	56	0.94 (0.90, 0.96)		
Two or more	22	0.96 (0.92, 0.98)		
None/not reported	16	0.95 (0.89, 0.98)		
**Engagement methods**			0.05	3.18 (< 0.001)
None	85	0.94 (0.91, 0.96)		
At least one engagement method	9	0.98 (0.94, 0.99)		

The median overall sample size was 349.5 (inter-quartile range, IQR: 139 to 1000). The median retention was 0.92 (IQR: 0.83 to 0.98). A random-effects meta-analysis indicated high heterogeneity between trials (τ^2^ = 3.38, *I*^2^ = 86.56%). However, the meta-regression analyses showed that several trial-level explanatory variables were found to partially explain the heterogeneity (Table 1).

In a sensitivity analysis, the trial with the lowest retention (0.42) [32] was removed and the random-effects meta-analysis point estimates were similar (0.95, 95% CI 0.93 to 0.96) with τ^2^ = 3.29, *I*^2^ = 86.26%.

### Trial context factors

#### Funding

Trials were mostly funded by the government, which included hospitals, healthcare settings, or research bodies such as the NIHR. Twenty-four trials were reported as being industry funded and 11 trials were funded from more than one funder which were often a collaboration between government and industry. There was evidence of an effect of funding (likelihood ratio test *p*-value 0.04, Table 1) where trials which were funded by multiple funders or third-sector (charity) funders had the highest estimated retention (0.98, 95% CI 0.94 to 0.99, Table 1).

#### Population, ICD-10 disease area, and duration of condition

Eighteen International Statistical Classification of Diseases and Related Health Problems (ICD)-10 2019 disease areas were represented, with the lowest retention for trials in the mental and behavioural disorders. There was no evidence of an effect of disease area on retention. Sixty-eight trials recruited participants from a clinical population (group of participants with a specific condition) and 26 from the general population. The duration of the condition which was under treatment in the trial was reported for 61 trials as chronic and in 33 trials as acute, with no evidence of a difference in retention between these two groups of trials. There was no evidence of an effect on retention.

#### Intervention aim

Nearly half of the trials aimed to manage the health condition of the participants. Thirty-four were preventative, which included trials to prevent a secondary condition developing other than the initial clinical diagnosis, and 16 trials aimed to cure a condition. There was no evidence of an effect on retention.

### Trial design factors

#### Trial design

Eighty-one trials involved participants from multiple sites and 13 were single sites. 82 trials were individually- and 12 cluster-randomised. 91 trials were designed as parallel-group trials and three trials were cross-over trials. 82 trials had two treatment groups, and most trials (*n* = 70) had less than 1000 participants randomised in total. Thirty per cent of trials with over 1000 patients randomised retain less than 80% of their participants, compared with 20% of trials with fewer than 1000 patients randomised. There was no evidence of an effect of these factors on retention.

#### Treatments

Most of the treatments in these trials were pharmacological (64 trials). There was no evidence of an effect on retention. There was evidence that the retention differed between those that had an active control treatment (0.97, *n* = 34), treatment-as-usual (0.92, *n* = 24) and placebo (0.94, *n* = 33), *p*-value 0.05 (Table 1). As the model would not converge when the wait-list-control trials (*n* = 3) were included in this analysis, due to the limited number of trials within this category, they were excluded and therefore the total number of trials was 91.

In a post-hoc sensitivity analysis, retention was investigated within the 33 placebo-control treatment groups only, in order to explore whether associations identified between retention and design factors may be confounded by treatment effects. The heterogeneity of the trials remains high (τ^2^ = 1.79), though is considerably lower than when synthesising all studies (τ^2^ = 3.29) irrespective of treatment. This suggests that retention may be associated with receiving an active treatment. Within placebo-control arms, there was no evidence of any association between retention and any of the trial design factors. However, restricting analyses to placebo-control arms only will have resulted in lower statistical power to detect associations with design factors, which may explain our results.

#### Participants

Fifty-eight trials included children aged from birth, and only six included children aged 11 to 17. There was evidence of an effect of age on retention (*p*-value 0.04, Table 1) where trials with children aged 11 and over had the higher estimated retention (0.98, 95% CI 0.91 to 1.00) than those including children from birth (0.96, 95% CI 0.93 to 0.97).

Sixty-three trials included active participation from adults (such as reporting the primary outcome or administering the intervention, e.g. teachers within schools). However, 31 RCTs did not report any other participants being involved. There was evidence of an effect of additional participants on retention (*p*-value 0.04, Table 1), but not in the direction expected. Trials which did not report including additional participants had the highest estimated retention, 0.97 (95% CI 0.94 to 0.98) whereas those including adults had a retention estimate of 0.93 (95% CI 0.90 to 0.96). It was not possible to explore whether there was any benefit to trials involving additional participants in the oldest age group, as only two out of those six trials involved additional participants.

#### Intervention setting and duration

Forty-three trials were of interventions carried out at home and 33 were within healthcare settings. Nearly 50% of trials had short interventions lasting 6 months or less. Thirty-nine trials were over 1 year, 28 were up to and including 6 months and 22 were between 6 and 12 months; therefore, the majority of RCTs lasted 1 year or less. There was no evidence of an effect on retention.

#### Follow-up

Most trials had five or more follow-up assessments over the course of the trial. However, six trials had only one follow-up assessment. Although this data includes assessments after the primary outcome it was felt that the follow-up intensity of the whole trial may influence the participant’s decision to remain in the trial. There was no evidence of an effect on retention.

Twenty-nine trials had primary outcome(s) reported up to and including 6 months, and 22 trials had their primary outcome reported between 6 and 12 months. There was evidence of an effect on retention (*p*-value 0.01, Table 1). The pattern of retention decreases with the length of time until the primary outcome is reported; 0.95 for up to 6 months (95% CI 0.91 to 0.97), 0.93 for 6 to 12 months (95% CI 0.86 to 0.97) and 0.90 for 1 year or over (95% CI 0.81 to 0.95). Those trials that had a variable time to the primary outcome (such as a time-to-event-outcome) had the highest retention (0.98, 95% CI 0.96 to 0.99).

There was evidence of an association between retention and the number of follow-up assessments which occurred before the primary outcome (*p*-value 0.01, Table 1). Thirty-five trials had five or more follow-up assessments before the primary outcome with 0.95 retained (95% CI 0.92 to 0.97). Nineteen trials did not have any follow-up assessments before the primary outcome, and the estimated retention was higher (0.92, 95% CI 0.84 to 0.96) than those which had one to four assessments (0.90, 95% CI 0.81 to 0.95).

We investigated whether there was an association between the length of trial and the number of follow-ups before the primary outcome within the trial. We found that there were similar proportions of trials lasting less than 6 months, which had no follow-ups (32%), or five or more follow-ups (31%), before the primary outcome. Whereas there were fewer shorter trials which had one to four follow-ups (17%). Trials which had either none or five or more follow-ups, also had high proportions of trial-specific clinic visits (63%, 68%), compared with trials with one to four follow-ups (54%) (Table 2).

**Table 2:** Number of follow-up visits before primary outcome by the length of trial, data collection method, and length of time to the primary outcome

	**Length of trial: less than 6 months**	**Primary data collection: trial-specific clinic visits**	**Length of time to the primary outcome: less than 6 months**	**Number of trials**
**Number of follow-up visits before the primary outcome**				
None	32%	68%	42%	19
One to four	17%	54%	21%	24
Five or more	31%	63%	31%	35

Fifty-five trials used a trial-specific clinic visit to collect their primary outcome, nine used a telephone call, nine used routine or hospital data, six used a research visit to participants, five used a survey without a visit, five were school-based visits, and five used other, or multiple, methods. There was evidence of an effect on retention (*p*-value 0.03, Table 1), with those that used hospital or routine data having the highest retention 0.99 (95% CI 0.97 to 1.00), followed by those that use other or multiple methods retention 0.97 (95% CI 0.86 to 0.99), telephone calls retention 0.95 (95% CI 0.86 to 0.98), trial-specific clinic visit retention 0.94 (95% CI 0.90 to 0.96).

Forty-one trials used a single outcome as the primary outcome. Most primary outcome(s) were reported using an objective measure (44 trials), defined as an outcome not calculated by a person such as blood pressure or glucose monitor. Five RCTs used routine data, or multiple methods (such as an objective measure as well as a self-reported outcome), to collect the primary outcome. Eleven trial’s primary outcome was reported by the additional participants (caregivers or teachers). Most trials did not report the use of more than one follow-up method during the trial. There was no evidence of an effect on retention.

#### Engagement methods

Eighty-five trials did not report any use of engagement methods to encourage participants during the trial. There was evidence that estimated retention increased from 0.94 (95% CI 0.91 to 0.96) to at least 0.98 (95% CI 0.94 to 0.99) if at least one engagement method was used (*p*-value 0.05, Table 1). Nine trials that used engagement methods included three trials that reminded participants to complete follow-up by calling or sending text messages, three trials that used a monetary incentive (one that also reminded participants about follow-up), and three that used multiple methods (one trial used monthly telephone calls, one used active surveillance of weekly telephone calls, and one used multiple phone numbers for families and emergency contacts, scheduling calls and sending phone text reminders, allowing for electronic completion and centralising all follow-up procedures at the lead institution, and a study diary provided to caregivers to use as a note-taking tool). For those nine trials which used engagement methods to improve retention, the primary outcome was collected in two trials by calling participants, three trials used clinic visits, two trials used researchers visiting participants, one trial used school visits, and one trial used participants taking swabs at home. The frequency of contact with participants outside of contact required to administer follow-up was not reported in enough of the trial papers to be used in a meta-regression.

## Discussion

In this review, we have found that the source of funding, age of participants, inclusion of additional participants, length of time until primary outcome, number of follow-up assessments before the primary outcome, primary outcome data collection method, type of control treatment, and engagement methods to encourage participants were associated with retention.

The strengths of this review are that we used a pre-specified data extraction template, two reviewers double-coded the abstracts, a selection of full-text papers were double data-extracted, and all analyses, other than those denoted, were pre-specified.

The overall retention of participants for the primary outcome of trials in our review was high (median 92%, IQR: 83%, 98%), although similar to a recent review of trials funded by NIHR [23] (median 88%, IQR: 80%, 97%), and funded by the United Kingdom Health Technology Assessment Programme [22] (median 89%, IQR: 79%, 97%). Even with this high overall retention, and small sample sizes of trials, there was still an association with specific trial-design factors, therefore we believe this association may be even stronger across other paediatric trials. A comparative review of trials published in higher- and lower-impact factor journals [33] found that in trials publish in lower-impact journals are, on average, more likely to be at risk of bias compared with those in higher-impact journals. The median sample size within the trials was also high (*n* = 349), in comparison paediatric trials that were reported as completed in data extracted from the ISRCTN registry (https://www.isrctn.com/) on 19/03/21 had a median of 163 enrolled participants (IQR 51 to 780, *n* = 297). We acknowledge that within this review the trials may be selective, but we believe that the selectivity is around the types of trials that are reported in these journals. For example, there are few trials in mental health conditions or using patient-reported outcomes measures, which may be challenging conditions to achieve high retention. An extension of our research could be to investigate retention in a wider range of paediatric trials, for example, those funded by major grant-awarding funders which may limit weaknesses and high risk of bias.

However, although pre-specified in our data extraction template, very few of these published papers reported any strategies used to improve retention. Therefore, we recommend further evaluation of engagement methods within trials are carried out.

This systematic review found that joint-, or charity-, funded trials had high estimated retention. This could be because these trials are often a partnership between academics who are more involved in running the trial, and industry who may have more money available to support repeated contact for those that do not complete follow-up measures or attend visits. Industry-funded trials are potentially more selective about the participants they recruit through their inclusion/exclusion criteria, as they often investigate the efficacy of treatment rather than the effectiveness in a pragmatic trial. This could lead to less attrition, as participants may be more ideal rather than “real-world”, and may be more likely to adhere to follow-up procedures because of payment for taking part, or perceived potential benefit from a treatment that otherwise would not be available. Charity-funded trials are often set up as an academic partnership, and potentially due to the condition or collaborations with patient organisations, may include more engaged participants. This finding is in contrast to previous research, although not specifically paediatric RCTs, where Toerien et al. [21] found no association between funding and retention. Clinical research networks in the UK offer incentives to clinical partners, such as hospitals or general practices, to recruit participants [34] but the same incentives are not offered for retaining participants. Parkinson et al. [35] investigated incentives for retention for trial recruiters in a scoping review. They found evidence that performance pay can significantly improve activity [36], with larger effects seen when targeted payments are at the individual rather than site [37]. They also conclude that there are challenges if incentivisation is linked to a specific outcome, such as recruitment, as that may lead to re-direction of resources away from other key trial activities, such as retention. The role of the clinician in retaining participants in trials is under-researched, although there is evidence that the use of motivational interviewing techniques in initial interactions with participants does improve retention to treatment or follow-up in some trials of weight loss [38], asthma [39] and substance abuse [40].

Unlike reviews of trials across all ages, we did not find any association between retention and size of trial [20, 22], number of treatment groups [21], or trial setting [22].

Similarly to Toerien et al. [21], we have found no evidence that the number of sites or treatment focus influenced retention. However, unlike Toerien et al. [21], Walters et al. [22] (review of trials funded and published by the UK Health Technology Assessment Programme, 2004–April 2016) and Jacques et al. [23] (review of trials published in the NIHR Journals Library, 1997–2020), we found evidence that an active or placebo control treatment had higher retention than treatment-as-usual. This may be because participants feel more involved in a trial with an active or placebo treatment, or may think that treatment-as-usual is inferior to the “new” intervention treatment. This has been termed “resentful demoralisation” where participants no longer wish to take part as they feel disappointed with their allocation to the control treatment [41]. A systematic review and meta-analysis of partially-randomised patient preference trials published between January 2015 and October 2018 compared retention within trials [42]. They found that in comparison with the cohort of participants who choose their treatment, those that were randomised, were less likely to be retained and more likely to crossover to other randomised treatment groups (relative risk percentage of participants lost to follow-up 1.3, 95% CI 1.0 to 1.6, *p*-value 0.03).

The age of participants also was associated with retention, with those trials which included the oldest children (aged 11 years old and over), and those that included the widest age-range (babies and over) having the highest estimated retention with narrow confidence intervals. Robinson et. al. found four RCTs in their systematic review [19] (28 RCTs, children from infancy to twelve years of age) that investigated the association between age and retention to final assessment, with only two trials showing evidence that younger children were less likely to be retained. We believe this may because older children are more likely to self-complete outcome measures, and caregivers may find it easier to attend follow-up assessments with older children. Unfortunately, we were unable to analyse these trial-level data more thoroughly due to the variation in age ranges reported across trials.

The trials in this review will have included those where the children were withdrawn by their carers/teachers, as well as those where the young people were able to withdraw or be lost-to-follow-up without others’ knowledge. This may mean that the retention of younger children in some trials may have similar patterns and associations with trial factors, to the retention of adults in trials. However, we feel that paediatric trials are different enough from those in adults, based on the involvement of, and potential data collection, from multiple participants, and the relative contribution of these different parties to a young person’s retention in a trial is usually unclear.

There also seemed to be evidence that having additional participants involved in the trials was associated with reduced retention. We think this could be due to additional participants, such as parents or teachers being asked to contribute significantly in the trial, such as completing proxy or health economic outcome measures, but the trial being of limited personal benefit. This may be especially challenging in trials which take place in schools. The potential impact of the inclusion of additional participants is the increase in costs associated with retention methods required with multiple participants. An alternative explanation could be a lack of reporting in those trials where we were unable to find any mention of additional participants. There may be an association between the age of the participant and the involvement of additional participants; however, we were unable to investigate this further. There is a paucity of evidence on how to involve additional participants, and how they can contribute to the retention of young people in paediatric trials.

Higher estimates of retention were seen for trials with more follow-up assessments that occurred before the primary outcome and those that had a shorter length of time until the primary outcome. A finding also seen by Karlson et al. [43] and Toerien et al. [21]. We found that trials with no follow-ups, or five or more follow-ups, before the primary outcome were often shorter, and the primary outcome collected at a clinical visit, compared with trials with one to four follow-ups. We also believe retention is unlikely to be affected by the number of follow-up assessments explicitly, but because trials which remain in regular contact with their participants maintain a higher level of engagement with trial follow-up. This finding is supported by qualitative research, where the way researchers interacted with and supported participants to attend follow-up visits by accommodating personal requirements, facilitated a sense of commitment to the study [1, 44]. Therefore, this result should not be judged in isolation, and retention to trials is likely to be influenced by a combination of factors. However, we found no evidence that trials with more follow-up assessments adversely affected retention, which may encourage trialists who are concerned about participant burden. We also found that higher retention was seen for those trials where the primary outcome data collection method required less active participation such as using routine or hospital data, or telephone calls, rather than attendance at trial-specific clinic visits.

Currently, there is no high-quality evidence for methods to improve retention as identified in the 2021 Cochrane systematic review of randomised retention strategies [11], and the systematic review of non-randomised retention strategies [12]. We found there was limited reported use of participant engagement methods such as incentives or reminders in trials. In the UK, NIHR guidance suggests that trials should consider appropriate payments for participation in research, as well as reimbursements for travel and subsistence [45]. Two recent systematic reviews found evidence that a monetary incentive compared with none within an RCT improved retention although the majority of included studies tested these incentives on the return of questionnaires, postal or online, rather than attending clinical visits. However, the evidence from these reviews lacked certainty and needs replication [11, 12].

We want to highlight, as others have, of the importance of evaluating retention initiatives through the use of a study within a trial (SWAT), and direct readers to the SWAT repository (https://www.qub.ac.uk/sites/TheNorthernIrelandNetworkforTrialsMethodologyResearch/SWATSWARInformation/Repositories/SWATStore/), the PROMETHEUS programme, who are funded to support researchers who wish to embed a SWAT within an RCT at no cost and highlight SWATs which are of priority (https://www.york.ac.uk/healthsciences/research/trials/swats/prometheus/), and the Trial Forge collaboration (www.trialforge.org), who are gathering evidence and designing of SWATs for implementation in RCTs.

Further qualitative research is needed into how the design of, and processes within, paediatric RCTs influence retention.

### Limitations

A potential limitation is that we investigated retention in 94 trials published in only six high-impact factor journals, and due to the limited variation in retention rates, associations between retention and trial-level factors may not be seen. We were only able to explore univariate relationships due to limited statistical power, and cannot rule out associations that may be seen with a larger sample size, combined associations, or confounding of factors influencing participant retention. A limitation of the data is not all trials reported involving additional participants, or engagement methods, and very few reported specific measures used to improve retention. Therefore, we are unable to suggest specific methods for trialists looking to improve retention in ongoing trials. We were also unable to analyse the wait-list-control trials due to the few trials within that category. A limitation of the analysis is that some meta-regressions were of aggregate characteristics (e.g. age), and any conclusions regarding the impact of these on retention at the individual level may suffer from ecological bias.

## Conclusion

This review suggests evidence of an association between retention in paediatric RCTs and source of funding, age of participants, inclusion of additional participants, length of time until primary outcome, primary outcome data collection method, number of follow-up assessments, type of control treatment, and engagement methods to encourage participation. Trials may be able to reduce attrition by including multiple, regular follow-ups with participants, specifically focusing on follow-ups before the primary outcome. Those designing trials also need to consider the use of appropriate engagement methods; however, we cannot suggest any specific engagement methods due to the limited reporting of methods to improve retention in included trials. This review has shown the unique challenge of retaining multiple participants (young people, and adults) in paediatric trials. Further qualitative research is required to investigate how this multi-participant retention can be improved, and how to encourage young people to remain involved.

## Supplementary Information

**Additional file 1:** **Appendix 1.** Search strategy. **Appendix 2.** Data extraction proforma. **Appendix 3.** Protocol.

**Additional file 2:** Data extracted.

## Data Availability

All data generated or analysed during this study are included in this published article and its supplementary information files.

## Abbreviations

RCT: Randomised controlled trial
NEJM: *New England Journal of Medicine*
BMJ: *British Medical Association*
JAMA: *Journal of the American Medical Association*
GLMM: Generalised linear mixed model
PRISMA: Preferred Reporting Items for Systematic reviews and Meta-Analyses
IQR: Inter-quartile range
95% CI: 95% Confidence interval
RoR: Research on Research
NIHR: National Institute for Health Research
ICD: International Statistical Classification of Diseases and Related Health Problems
SWAT: Study within a trial
CONSORT: Consolidated Standards of Reporting Trials
