# Supplementary material for: Participant retention in paediatric randomised controlled trials published in six major journals 2015–2019: systematic review and meta-analysis
Source: Trials. 2023 Jun 14;24:403. doi: 10.1186/s13063-023-07333-w (PMC10265847; doi:10.1186/s13063-023-07333-w)
Supplement: Supplementary file 1 — Additional file 1: Appendix 1. Search strategy. Appendix 2. Data extraction proforma. Appendix 3. Protocol. [file 13063_2023_7333_MOESM1_ESM.docx]

**Appendix 1: Search strategy**

random* control* trial* OR RCT*) AND ("2015/01/01"[PDat]: "2019/12/31"[PDat]) AND (child[MeSH] OR adolescent[MeSH]) AND ("The New England journal of medicine"[Journal] OR "British medical journal"[Journal] OR "JAMA"[Journal] OR "Lancet (London, England)"[Journal] OR “Pediatrics"[Journal] OR "JAMA pediatrics"[Journal])

**Appendix 2: Data extraction proforma**

| **Data extraction element** | **Categories and definitions** |
| --- | --- |
| Title | Free-text |
| First author or trial team | Free-text |
| ICD-10 2019 disease area | <https://icd.who.int/browse10/2019/en> |
| Journal | Options: NEJM, BMJ, JAMA, Lancet, Pediatrics, JAMA pediatrics |
| Funding source | Options: academic, government, third sector, industry, other (free text)  *If university-funded/research department affiliation of trial authors; then academic funded. If no funding/not clear report as other. Industry; only if involved in designing/administering trial. Third sector is charity.* |
| Severity of condition | Options: Chronic, acute, preventative, other (free text)  *If participants have a chronic condition, mark Severity as chronic. If preventive; what was the severity of the condition the trial was trying to prevent. Example: language-delay treatment denoted as preventative.* |
| Population | Options: clinical, general, other (free text)  *Where were participants recruited from e.g., recruitment from secondary services or a clinical cohort; then clinical. If pre-clinical i.e., preventive of specific disease; define as general.* |
| Sites | Options: multi, single, other (free text) |
| Description of population (e.g., socio-deprivation or ethnicity) | Free text  *e.g., age, gender, ethnicity, socio-economic status, or caregiver factors such as educational attainment. Trial recruitment geographical area.* |
| Rational of trial | Options: preventative, management of condition, curative, other (free text) |
| Trial design | Options: parallel group, cross-over, stepped-wedge, adaptive, other (free-text)  *Cross-over trial: length of intervention includes the control + intervention + washout period.* |
| Randomisation | Options: individual, cluster, other (free text) |
| Age range of participants | Options: Babies (under 2), pre-school (2-4), primary (5-11), pre-teenager (12-13), teenager (13-16), adolescent (16-18), other (free text).  *Age at randomisation or, if not reported, recruitment. If the ages of the participants spanned more than one group, the age range was reported in other.* |
| Additional participants | Options: parents/carer, teachers, siblings, family, none, other (free text)  *Only reported if the additional participants had to do more than give consent i.e., they responded to questionnaires or administered intervention e.g., teachers within schools. If multiple other participants, report all in other.* |
| Intervention setting | Options: home, primary care, secondary care, tertiary care, third sector, school, other (free text)  *Where was the intervention administered e.g. ointments applied to children or monitoring glucose levels at home.* |
| Length of intervention | Options: in-hospital stay, between 1 to 3-months, over 3 to 6-months, over 6 to 12-months, one year, other  *Also includes any on-going training or motivational messages delivered by trial team* |
| Length of trial for participant | Options: in-hospital stay, up to and including 6-months, over 6 to 12-months, one year, up to and including two years, up to and including three years, more than 3 years, other (free-text)  From time of randomisation to final follow-up. |
| Total number of follow-ups in trial | Options: one, two, three, four, five or more, time-to-event, other (free-text)  *Definition of follow-up includes any data collected on any participants; either self-reported or collected by researchers e.g., telephone calls*  *If it can be found for trials with time to event primary outcomes report how many follow-ups were in the planned follow-up period. If not possible, just report as time-to-event.* |
| Time since randomisation to primary outcome | Options: time to first event, up to and including 6-months, over 6 to 12-months, at one year, over 1 year, other (free-text)  *If multiple primary outcomes; time from randomisation to final outcome timepoint* |
| Number of follow-ups before primary outcome | Options: none, one two, three, time to event, other (free text)  *If it can be found for trials with time to event primary outcomes report how many follow-ups were before the primary outcome. If not possible, just report as time-to-event.* |
| Primary outcome data collection method | Options: online survey/website, paper based, telephone call, smartphone/tablet application, electronic device, home visit, clinic visit, routine data, other (free text)  *How was the participant asked to contribute to the primary outcome; what action did they have to take. If they had to attend a visit either at home or clinic, this should be completed as a visit. A clinic visit includes anything that was a clinical assessment. If the location of the visit is unclear, or participants were given a choice mark as other and describe. Paper-based is completing and returning a questionnaire. Electronic device e.g., accelerometer or glucose monitor.* |
| Number of observations that went into the primary outcome | Options: single, repeated measures over time, time-to-event, composite.  *Definition of repeated measures over time – where the primary outcome measure was collected more than once. Composite includes multiple primary outcomes.* |
| Frequency of contact between trial and participants | Options: As follow-up, other (free text) |
| Participant engagement methods | Options: No, other (free text)  *Anything over and above outcome data collection. Report whether in one or multiple trial groups.* |
| Primary outcome reported by whom | Options: participant self-report, parent/carer, teacher, health care practitioner (HCP), objective measurement, routine data, other (free text)  *Objective measurement – anything measured not by a person e.g., blood pressure cuff or glucose monitor. HCP also includes the trial team.* |
| Other methods of follow-up | Options: online survey/website, paper-based, telephone call, smartphone/table application, electronic device, home visit, clinic visit, routine data, other (free-text). |
| Are results presented by  missingness? | Options: no, other (free text)  *Describe any attempts to summarize why results were not possible for all participants e.g., baseline characteristics by missing outcome data or if the authors carried out any missing data sensitivity analysis.* |
| Intervention type | Options: pharmacological, medical device, surgical procedure, psychological therapy, behavioural change, other  *Behaviour change e.g., physical activity, handwashing, educational resources.* |
| Treatment 1 (control) | Free text |
| Control | Options: treatment-as-usual, wait-list control, active, sham, placebo, conservative management, other (free-text) |
| Treatment 1 primary outcome completion rate | *cluster randomized trials: only include participants from when they consented, as some may have declined or not been eligible.*  *May not just be those that were included in the primary analysis. Need to check, if possible. Even if participants dropped out of trial, they may have contributed primary outcome data such as through routine data collection. If possible, do not count as completing the primary outcome those whose missing primary outcome was imputed. If it is not possible to work out why participants were excluded from reporting the primary outcome (e.g., industry trial: excluded because of lack of adherence to treatment), report the number that were analysed and make a note. If participants are reported as died before primary outcome, include them as responding to primary outcome but make a note that xx died.* |
| Treatment 2 (intervention) | Free text |
| Treatment 2 primary outcome completion rate | As above |
| Treatment 3 | Free text |
| Treatment 3 primary outcome completion rate | As above |
| Treatment 4 | Free text |
| Treatment 4 primary outcome completion rate | As above |

**Appendix 3: Protocol**

Aim: To explore the rate of retention in paediatric RCTs

Search terms:

random* control* trial* OR RCT*) AND ("2015/01/01"[PDat]: "2019/12/31"[PDat]) AND (child[MeSH] OR adolescent[MeSH]) AND ("The New England journal of medicine"[Journal] OR "British medical journal"[Journal] OR "JAMA"[Journal] OR "Lancet (London, England)"[Journal] OR “Pediatrics"[Journal] OR "JAMA pediatrics"[Journal])

Database: MEDLINE

Journals: The four medical journals with the highest impact factor will be searched (The New England journal of medicine, British medical journal, JAMA and Lancet) as well as two paediatric journals with the highest impact factor (Pediatrics and JAMA paediatrics). These journals were chosen using impact factor data from 2018.

Inclusion criteria

- Randomised controlled trial
- Children aged under 18
- Intervention targeted at children/adolescents
- Reports retention rate of participants
- If an article includes adult and children/adolescents, the completion data needs to be presented separately for children/adolescents

Exclusion criteria

- Only adults aged 18 or over
- Systematic review
- Meta-analysis
- Intervention targeted at parents/carers or teachers
- N-of-1 trials
- Follow-up trials to original RCT
- Conference abstracts
- Commentary on original RCT

Review software: Covidence will be used to screen, sort and store all papers that are found.

Methodology: The titles and abstracts will be reviewed independently by one other researcher.

If there are less than 250 articles chosen for full text review (approximately 50 RCTs/year, over 5 years) all articles will be used. If not, a stratified random sample within each journal of those meeting the inclusion criteria at abstract review will be taken based on the overall number of RCTs per year.

Data will be extracted from each RCT including ages of participants, medical condition, length and type of intervention (medical, therapy, surgical), length of trial and follow-up, completion rate of primary outcome, time-point at which the primary outcome(s) is measured, how long after treatment conclusion the primary outcome time-point occurs, how follow-up was conducted (e.g. paper-based, online, use of apps) and what participant engagement methods were used by the trial (e.g. newsletters) (Appendix 2).

If multiple primary outcome(s) are collected the final primary outcome that is collected will be recorded. If the primary outcome is completed at hospital discharge this will be noted. As trials may use not use a patient-reported primary outcome, the completion of the PROMS will also be reported.

Analysis will be a qualitative description of retention and a quantitative comparison of the retention rate (defined as the completion rate of the primary outcome) between/within trials based on factors, as described above.

Some updates to this protocol that occurred after 10 papers were reviewed for consensus:

- Funding source: if university-funded/research department affiliation of trial authors; then academic funded. If no funding/not clear report as other. Industry; only if involved in designing/administering trial. Third sector is charity.
- Severity of condition: If participants have a chronic condition, mark Severity as chronic. If preventive; what was the severity of the condition the trial was trying prevent? Language delayed treatment denoted as preventative.
- Population: where were participants recruited from e.g. recruitment from secondary services or a clinical cohort; then clinical. If pre-clinical i.e. preventive of specific disease; define as general.
- Description of population: age, gender, ethnicity, socio-economic status, or parent/carer factors such as educational attainment.
- Additional participants: only include if they had to do more than give consent i.e., they responded to questionnaires or administered intervention e.g. teachers within schools
- Age range: age at randomisation or if not reported, recruitment.
- Intervention setting: where was the intervention administered e.g. ointments applied to children at home or monitoring glucose.
- Length of trial: from randomisation to final follow-up
- Total number of follow-ups in trial/number of FUs before primary outcome: If it can be found for trials with time to event primary outcomes report how many FU were in the planned FU period. If not possible, just report as time-to-event.
- Length of intervention: includes any on-going training or motivational messages delivered by trial team
- Follow-up includes any data collected on any participants; either self-reported or collected by researchers e.g. telephone calls
- If multiple primary outcome timepoints; time from randomisation is to final outcome timepoint
- Primary outcome data collection method: how was the participant asked to contribute to the primary outcome; what action did they have to take. If they had to attend a visit either at home or clinic, this should be completed as a visit. A clinic visit includes anything that was a clinical assessment. If the location of the visit is unclear where, or participants were given a choice (Azizi 2019), then mark as other and describe. Paper-based is completing and returning a questionnaire. Electronic device e.g. accelerometer or glucose monitor.
- Number of observations that went into the primary outcome: repeated measures over time – where the primary outcome was collected more than once and analysed over time
- Participant engagement methods: anything over and above outcome data collection. Report whether in one or multiple trial groups.
- Primary outcome reported by whom: objective measurement – anything measured not by a person e.g. blood pressure cuff or glucose monitor.
- Are results presented by missingness; describe any attempts to summarise why results were not possible for all participants e.g. baseline characteristics by missing outcome data or if the authors carried out any missing data sensitivity analysis.
- Behaviour activation definition included any behaviour change e.g. physical activity, handwashing, educational resources.
- Cluster randomised trials; only include participants from when they consented, as some may have declined or not been eligible.
- Number responding to primary outcome may not just be those that were included in the primary analysis. Need to check, if possible. Even if participants dropped out of trial, they may have contributed primary outcome data such as through routine data collection. If possible, do not count as completing the primary outcome those whose missing primary outcome was imputed. If it is not possible to work out why participants were excluded from reporting the primary outcome (e.g. in pharma trial: excluded because of lack of adherence to treatment), report the number that were analysed and make a note. If participants are reported as died before primary outcome, include them as responding to primary outcome but make a note that xx died.
- Cross-over trial: length of intervention includes the control + intervention + washout period.

Any discrepancies will be discussed and concluded by both full-text reviewers.

Analysis

Report descriptive statistics, such as number of trials, under the headings of trial context (e.g., funding, age range of participants) and trial design (e.g., number of follow-ups). Summarise the free-text questions e.g., results reported by missing data.

Compare the proportion of retention between the control and intervention groups (multiple interventions, combine as total proportion). Compare difference in proportions/risk ratios within categories of factor extracted, e.g., pharmacological interventions, behavioural change, and report as forest plots.

If no difference in proportion of retention between treatment groups, combine as total proportion across all treatment groups. Estimate the retention for each trial with its standard error, and compare pooled estimates between categories.
